# The Budding Yeast “*Saccharomyces cerevisiae*” as a Drug Discovery Tool to Identify Plant-Derived Natural Products with Anti-Proliferative Properties

**DOI:** 10.1093/ecam/nep069

**Published:** 2011-06-23

**Authors:** Bouchra Qaddouri, Abdelkarim Guaadaoui, Ahmed Bellirou, Abdellah Hamal, Ahmed Melhaoui, Grant W. Brown, Mohammed Bellaoui

**Affiliations:** ^1^Laboratoire de Génétique et Biotechnologies, Faculté des Sciences, Université Mohamed Premier, Oujda 60000, Morocco; ^2^Département de Biologie, Faculté des Sciences, Université Mohamed Premier, Oujda 60000, Morocco; ^3^Department of Biochemistry, University of Toronto, Toronto, ON, Canada M5S 1A8; ^4^Terrence Donnelly Centre for Cellular and Biomolecular Research, University of Toronto, Toronto, ON, Canada M5S 1A8; ^5^Faculté Pluridisciplinaire de Nador, Université Mohamed Premier, B.P: 300 Selouane 62700, Morocco

## Abstract

The budding yeast *Saccharomyces cerevisiae* is a valuable system to study cell-cycle regulation, which is defective in cancer cells. Due to the highly conserved nature of the cell-cycle machinery between yeast and humans, yeast studies are directly relevant to anticancer-drug discovery. The budding yeast is also an excellent model system for identifying and studying antifungal compounds because of the functional conservation of fungal genes. Moreover, yeast studies have also contributed greatly to our understanding of the biological targets and modes of action of bioactive compounds. Understanding the mechanism of action of clinically relevant compounds is essential for the design of improved second-generation molecules. Here we describe our methodology for screening a library of plant-derived natural products in yeast in order to identify and characterize new compounds with anti-proliferative properties.

## 1. Introduction

Despite an increasing awareness and expenditure of resources, the incidence of cancer has not declined in decades, and, in fact, is rising in some areas at an alarming rate. Despite enormous outlays of research dollars, the number of novel cancer drugs has remained flat over the past two decades. Screening natural products for potential new drugs represents the best avenue to discover new, inexpensive and effective drugs to treat cancer [1, 2].

Morocco is very rich in medicinal plants widely used in folk medicine to prevent or cure many diseases. Morocco's unique geography comprises all bioclimatic stages in the Mediterranean zone, with an extraordinarily abundant and diverse vegetation represented by *∼*42 000 plant species. Estimates of the number of medicinal plants used by Moroccan healers suggest that several hundreds of species containing several thousand bio-active compounds could be useful in treating human disease [3–8]. However, it is often difficult to identify these bioactive natural compounds and to study their effect at the cellular level or their mechanism of action. Therefore, understanding the role of these natural compounds requires the development of high resolution yet cost-effective biological assay systems.

The budding yeast *Saccharomyces cerevisiae* is an excellent model system for identifying and studying antifungal compounds because of the functional conservation of fungal genes. Therefore, to accelerate the discovery of bioactive compounds with antifungal activity, Gassner et al. [9] have developed an automated yeast toxicity screen which they call high-throughput (HT) yeast halo assay. Yeast studies have also contributed greatly to our understanding of the biological targets and modes of action of bioactive compounds [10–14]. Understanding the mechanism-of-action of clinically relevant compounds is essential for the design of improved second-generation molecules. Yeast-based functional genomics technologies are excellent means for mechanistic studies of clinically relevant compounds. Due to the highly conserved nature of the cell-cycle machinery between yeast and humans, the budding yeast is also an excellent model system to study cell-cycle regulation [15–17], which is defective in cancer cells. Therefore, yeast studies are directly relevant to anticancer-drug discovery [10, 18]. Here we show, through an example, our procedure for the identification and characterization of new plant-derived natural products with anti-proliferative properties using the budding yeast as a drug discovery tool.

## 2. Selection of Growth-Inhibitory Compounds

**Figure 1:**
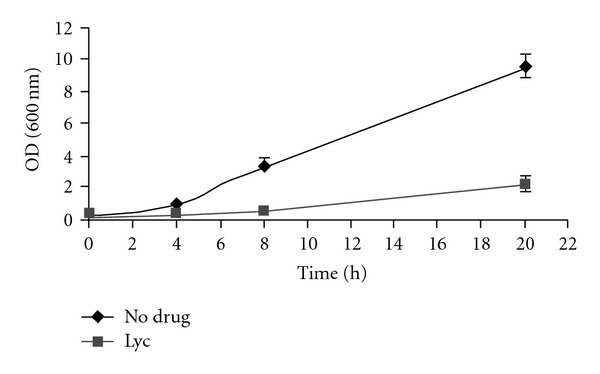
Growth of wild-type (OD 600 nm) as a function of time. Wild-type cells were diluted from an overnight culture to an OD600 of *∼*0.01 and allowed to grow until the OD600 reached *∼*0.05, ensuring that the cells were in logarithmic phase. Drug was then added and growth rate was measured as the optical density of cells (OD600) as a function of time (h) in rich medium. All compounds were diluted in 100% DMSO, and all assays, including the “no compound” control, contained 1% DMSO. The growth in the presence of 100 *μ*g/ml of natural compound Lyc is presented.

In order to identify new molecules of potential therapeutic interest, we first constructed a library of chemical molecules provided by phytochemists. We next assessed the growth-inhibition effects of these molecules in yeast to identify compounds with antifungal activity. Thus, from 40 plant extracts and 14 purified plant-derived natural molecules analyzed, we identified 14 plant extracts and 8 natural molecules that have clear growth-inhibitory effects. We show here the characterization of Lyc, a plant-derived natural alkaloid product. To assess the effect of Lyc on yeast growth, yeast cells in logarithmic phase were incubated with or without Lyc. As shown in Figure 1, addition of Lyc resulted in a significantly reduced growth rate of yeast cells, indicating that Lyc exhibited bioactivity in yeast, resulting in inhibition of growth. Using this screen we selected a number of growth-inhibitory compounds that were further characterized in order to understand their mode of action.

## 3. Mechanism of Action

### 3.1. Cell-Cycle Analysis

**Figure 2:**
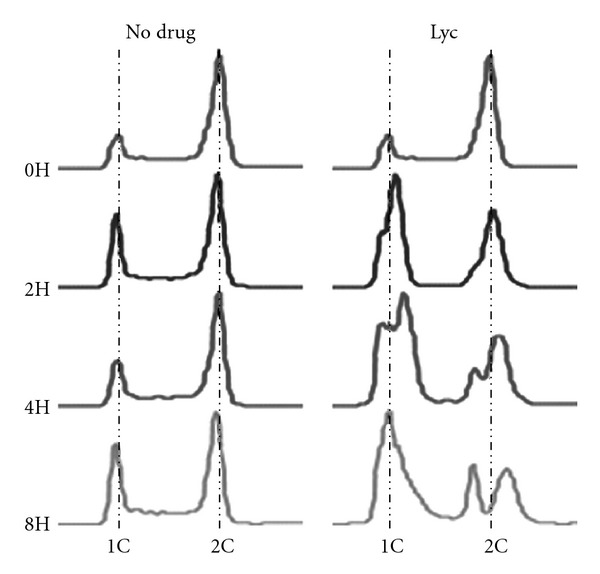
Cell-cycle analysis. In the presence of Lyc, cells show aberrant DNA content. Cells treated with Lyc display cell-cycle perturbation. Wild-type strain was incubated in the presence or absence of Lyc. Samples at the indicated time points were analyzed by flow cytometry to measure DNA content (1C and 2C peaks are indicated).

Cell-cycle analysis was first undertaken to determine whether a compound causes cell-cycle perturbation. In *S. cerevisiae*, it is easy to monitor cell-cycle progression by measuring DNA content using Fluorescence-Activated Cell Sorting (FACS) [18]. For example, abnormal accumulation of cells with 1C DNA content of an exponentially growing culture usually indicates a defect in the G1 phase of the cell cycle. Thus, to further investigate the growth defect caused by Lyc treatment, cell-cycle progression was assessed using FACS analysis. Logarithmically growing cultures were incubated in the presence or absence of Lyc. At the indicated times samples were fixed and the DNA contents of cells in each sample were measured by flow cytometry (Figure 2). In comparison with the no drug control culture, an increase in the number of cells with a 1C (or G1) DNA content was observed, starting at 2 h and persisting for the duration of the experiment. After 4 and 8 h of Lyc treatment, accumulation of cells with abnormal DNA contents (neither 1C nor 2C) was evident. Thus, these results suggest that Lyc perturb cells-cycle progression.

### 3.2. Phenotypic Analysis

**Figure 3:**
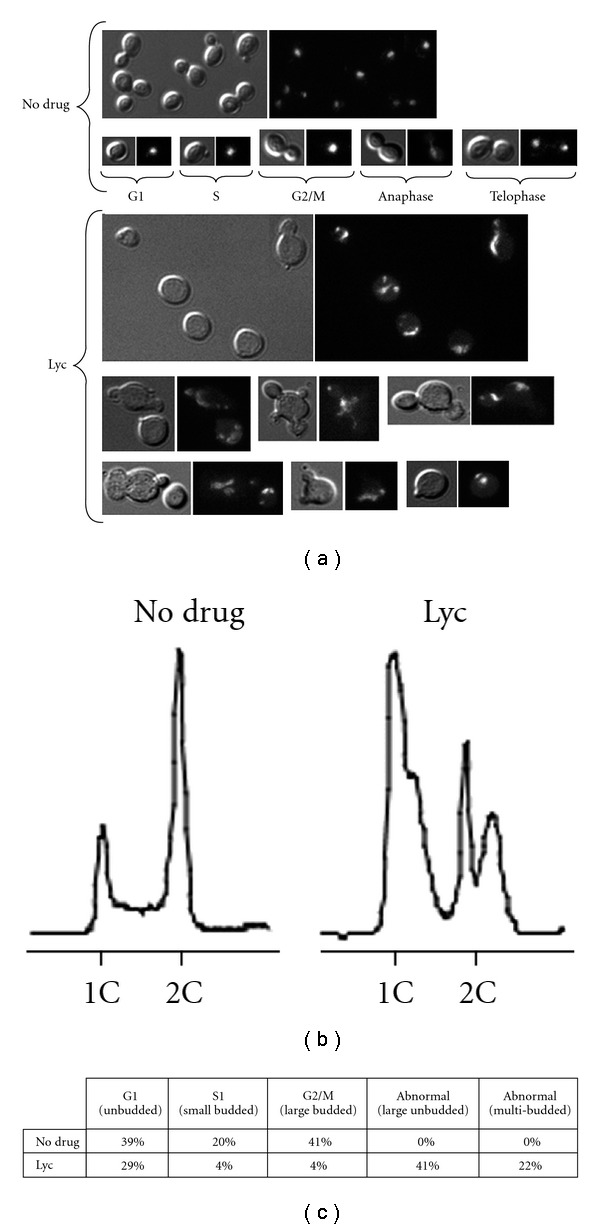
Phenotypic analysis. (a) Representative images of asynchronously growing wild-type cells in the absence or presence of Lyc for 8 h. Cells treated with Lyc exhibit strong morphological alteration and fragmented nuclei. For each field of cells, differential interference contrast (left) and DAPI staining of DNA (right) images were taken. Specific cell morphologies at different stages of the cell cycle are shown for the no drug control. (b) FACS profile of cells from the experiment in (a). (c) Budding index was measured for growing wild-type cells from the experiment in (a). Cells were counted in each of two experiments, and the average of the two experiments is showed.

*Saccharomyces cerevisiae* divides by budding and shows specific cell morphologies at different stages of the cell cycle. Unbudded cells are in the G1 phase, small budded cells are in the S phase, and large budded cells are in the G2/M phase of the cell cycle (Figure 3(a)). Thus, it is easy to monitor cell-cycle progression by simply observing cells using fluorescence and differential interference contrast microscopy. Therefore, to further investigate the effect of Lyc on cell-cycle progression, yeast cells in logarithmic phase were incubated with or without Lyc and then visualized by differential interference contrast (DIC) microscopy. We discovered that cells treated with Lyc exhibit a characteristic morphological alteration, with most cells either unbudded or abnormal with multiple buds (Figure 3(a)). Additionally, DAPI staining of cellular DNA revealed that treated cells typically had a fragmented nucleus (Figure 3(a)). Most cells had two or more DAPI-staining nuclear fragments, indicating that Lyc disrupts the integrity of the nucleus, consistent with the accumulation of cells with abnormal DNA contents observed by FACS analysis of the same cell sample (Figure 3(b)). Furthermore, we scored the proportion of unbudded (G1), small budded (S), large budded (G2/M) and abnormal cells in the presence or absence of Lyc. As shown in Figure 3(c) and in comparison with the no drug control, most cells treated with Lyc were abnormal (41% large unbudded and 22% multi-budded cells) and only few cells were in S or G2/M phases of the cell cycle. Together, these data suggest that treatment with Lyc impairs normal cell-cycle progression, perhaps as a result of nuclear fragmentation. However, for more detailed analysis of the effect of Lyc on cell-cycle progression, cells should be arrested in specific stages of the cell cycle and then released synchronously into the cell cycle [19–21]. To detect G1 or S phase progression defects, cells can be arrested in G1 with the mating pheromone alpha factor, then released into media with or without Lyc. In contrast, mitotic defects can be detected following arrest in G2 using the microtubule inhibitor nocodazole, then released into media with or without Lyc.

### 3.3. Chemical-Genetic Profiling

Yeast-based functional genomics technologies are excellent means for mechanistic studies of compounds with antifungal activity. In particular, chemical-genetic profiling has been used to study the mode of action of a number of growth-inhibitory compounds. Recently, in a proof-of-concept manuscript, we demonstrated that chemical-genetic profiling in yeast contributes greatly to understand the modes of action of bioactive compounds in human cells [10]. Briefly, in these assays, a pooled collection of homozygous (or *MATα* haploid) viable deletion mutants, each in a different gene, is first grown in the presence or absence of a growth-inhibitory compound. Then, following growth, deletion strains that are under-represented, and therefore sensitive, in the presence of compound relative to the control condition are identified by hybridizing the unique DNA sequences which flank each deletion to their complements on a DNA microarray [10–14]. This system identifies a set of deletion mutant strains hypersensitive to a given compound, referred to as the chemical-genetic profile. The results of these studies typically determine which pathways are important for drug resistance and therefore give insights into compound mode-of-action. In order to identify the drug target chemical-genetic profiling is carried out using a complete collection of heterozygous deletion strains. In this second type of profiling, referred to as HaploInsufficiency Profiling (HIP), a pooled collection of all *∼*6000 heterozygous deletion mutants, each in a different gene, can be grown in the presence or absence of a drug. Then, strain fitness can be determined by DNA microarray analysis of the PCR-amplified barcodes incorporated into each strain. Heterozygous strains most sensitive to drug carry deletions in genes which encode the likely drug targets [11].

Therefore, in order to determine the mode of action of Lyc chemical-genetic profiling was performed using the complete pool of barcoded *MATα* haploid viable deletion strains. Preliminary data show that the most sensitive mutants to Lyc are involved in translation regulation, suggesting that this compound may affect protein biosynthesis. Further investigation is required to confirm this result and to determine the precise target of Lyc.

## 4. Conclusion

The budding yeast *S. cerevisiae* is an excellent model system for identifying plant-derived natural products with anti-proliferative properties. As proof of principle, we showed that yeast cells treated with Lyc exhibited cell-cycle perturbation, fragmented nuclei, and morphological alterations. Compounds with these properties are excellent candidates for further study, as they might target components of the cell-cycle regulation machinery, and so could have anti-proliferative properties of therapeutic value. Such compounds could be useful in treating proliferative diseases such as cancers, or in treating fungal infections.

## References

[1] Newman DJ, Cragg GM (2007). Natural products as sources of new drugs over the last 25 years. *Journal of Natural Products*.

[2] Cooper EL (2004). Drug discovery, CAM and natural products. *Evidence-Based Complementary and Alternative Medicine*.

[3] Samane S, Noël J, Charrouf Z, Amarouch H, Haddad PS (2006). Insulin-sensitizing and anti-proliferative effects of Argania spinosa seed extracts. *Evidence-Based Complementary and Alternative Medicine*.

[4] Merzouki A, Ed-derfoufi F, Molero Mesa J (2000). Contribution to the knowledge of Rifian traditional medicine. II: Folk medicine in Ksar Lakbir district (NW Morocco). *Fitoterapia*.

[5] Jouad H, Haloui M, Rhiouani H, El Hilaly J, Eddouks M (2001). Ethnobotanical survey of medicinal plants used for the treatment of diabetes, cardiac and renal diseases in the North centre region of Morocco (Fez-Boulemane). *Journal of Ethnopharmacology*.

[6] Eddouks M, Maghrani M, Lemhadri A, Ouahidi M-L, Jouad H (2002). Ethnopharmacological survey of medicinal plants used for the treatment of diabetes mellitus, hypertension and cardiac diseases in the south-east region of Morocco (Tafilalet). *Journal of Ethnopharmacology*.

[7] El-Hilaly J, Hmammouchi M, Lyoussi B (2003). Ethnobotanical studies and economic evaluation of medicinal plants in Taounate province (Northern Morocco). *Journal of Ethnopharmacology*.

[8] Tahraoui A, El-Hilaly J, Israili ZH, Lyoussi B (2007). Ethnopharmacological survey of plants used in the traditional treatment of hypertension and diabetes in south-eastern Morocco (Errachidia province). *Journal of Ethnopharmacology*.

[9] Gassner NC, Tamble CM, Bock JE (2007). Accelerating the discovery of biologically active small molecules using a high-throughput yeast halo assay. *Journal of Natural Products*.

[10] Yu L, Lopez A, Anaflous A, El Bali B, Hamal A, Ericson E (2008). Chemical-genetic profiling of imidazo[1,2-*a*]pyridines and -pyrimidines reveals target pathways conserved between yeast and human cells. *PLoS Genet*.

[11] Giaever G, Shoemaker DD, Jones TW (1999). Genomic profiling of drug sensitivities via induced haploinsufficiency. *Nature Genetics*.

[12] Giaever G, Flaherty P, Kumm J (2004). Chemogenomic profiling: identifying the functional interactions of small molecules in yeast. *Proceedings of the National Academy of Sciences of the United States of America*.

[13] Lum PY, Armour CD, Stepaniants SB (2004). Discovering modes of action for therapeutic compounds using a genome-wide screen of yeast heterozygotes. *Cell*.

[14] Parsons AB, Lopez A, Givoni IE (2006). Exploring the mode-of-action of bioactive compounds by chemical-genetic profiling in yeast. *Cell*.

[15] Chang M, Bellaoui M, Charles-Boone C, Brown GW (2002). A genomewide screen for methyl methanesulfonate sensitive mutants reveals genes required for S phase progression in the presence of DNA damage. *Proceedings of the National Academy of Sciences of the United States of America*.

[16] Bellaoui M, Chang M, Ou J, Xu H, Boone C, Brown GW (2003). Elg1 forms an alternative RFC complex important for DNA replication and genome integrity. *EMBO Journal*.

[17] Chang M, Bellaoui M, Zhang C, Desai R, Morozov P, Delgado-Cruzata L (2005). *NCE4*, a suppressor of genomic instability, encodes a member of the RecQ Helicase/Topo III complex. *EMBO Journal*.

[18] Hartwell LH (2002). Nobel Lecture: yeast and cancer. *Bioscience Reports*.

[19] Haase SB, Lew DJ (1997). Flow cytometric analysis of DNA content in budding yeast. *Methods in Enzymology*.

[20] Jacobs CW, Adams AE, Szaniszlo PJ, Pringle JR (1988). Functions of microtubules in the Saccharomyces cerevisiae cell cycle. *Journal of Cell Biology*.

[21] Breeden LL (1997). *α*-Factor synchronization of budding yeast. *Methods in Enzymology*.

